# Building evidence on HRH programme implementation: assessment in 15 Latin American and Caribbean countries

**DOI:** 10.1186/1472-6963-14-S2-O34

**Published:** 2014-07-07

**Authors:** Mario Roberto Dal Poz

**Affiliations:** 1 Universidade do Estado do Rio de Janeiro, Brazil

## 

The health systems in the Americas region are characterized by fragmentation and segmentation that constitute an important barrier for expanding coverage, achieving integrated primary health care, and reducing the inefficiency and discontinuity of care.

An assessment of the HRH programmes that have been implemented at country level was developed as part of the measurement of the 20 Human Resources for Health (HRH regional goals 2007-2015 adopted in 2007 the Pan-American Sanitary Conference (CSPA)).

The exercise was a combination of academic research and the development/application of an advocacy tool, involving policy makers and stakeholders to influence decision-making on the development, implementation or change the HRH programmes, while building evidence through a structured approach based on qualitative and quantitative information, and exchange and dissemination of best practices.

The presentation will cover the methodological challenges, as well as a summary of the main findings of the study that included 15 countries: El Salvador, Costa Rica, Guatemala, Honduras, Nicaragua, Panama, Dominican Republic and Belize in Central America, Ecuador, Colombia, *Chile* and Peru in the Andean region and Argentina, Paraguay and Uruguay in the Mercosur.

